# Molecular and Cellular Heterogeneity in Rheumatoid Arthritis: Mechanisms and Clinical Implications

**DOI:** 10.3389/fimmu.2021.790122

**Published:** 2021-11-25

**Authors:** Jianan Zhao, Shicheng Guo, Steven J. Schrodi, Dongyi He

**Affiliations:** ^1^ Guanghua Clinical Medical College, Shanghai University of Traditional Chinese Medicine, Shanghai, China; ^2^ Department of Rheumatology, Shanghai Guanghua Hospital, Shanghai University of Traditional Chinese Medicine, Shanghai, China; ^3^ Computation and Informatics in Biology and Medicine, University of Wisconsin-Madison, Madison, WI, United States; ^4^ Department of Medical Genetics, School of Medicine and Public Health, University of Wisconsin-Madison, Madison, WI, United States; ^5^ Arthritis Institute of Integrated Traditional and Western Medicine, Shanghai Chinese Medicine Research Institute, Shanghai, China

**Keywords:** rheumatoid arthritis, heterogeneity, pathophysiology, interaction, genetics, mechanism, precision medicine, responsiveness

## Abstract

Rheumatoid arthritis is an autoimmune disease that exhibits significant clinical heterogeneity. There are various treatments for rheumatoid arthritis, including disease-modifying anti-rheumatic drugs (DMARDs), glucocorticoids, non-steroidal anti-inflammatory drugs (NSAIDs), and inflammatory cytokine inhibitors (ICI), typically associated with differentiated clinical effects and characteristics. Personalized responsiveness is observed to the standard treatment due to the pathophysiological heterogeneity in rheumatoid arthritis, resulting in an overall poor prognosis. Understanding the role of individual variation in cellular and molecular mechanisms related to rheumatoid arthritis will considerably improve clinical care and patient outcomes. In this review, we discuss the source of pathophysiological heterogeneity derived from genetic, molecular, and cellular heterogeneity and their possible impact on precision medicine and personalized treatment of rheumatoid arthritis. We provide emphasized description of the heterogeneity derived from mast cells, monocyte cell, macrophage fibroblast-like synoviocytes and, interactions within immune cells and with inflammatory cytokines, as well as the potential as a new therapeutic target to develop a novel treatment approach. Finally, we summarize the latest clinical trials of treatment options for rheumatoid arthritis and provide a suggestive framework for implementing preclinical and clinical experimental results into clinical practice.

## Introduction

Rheumatoid arthritis (RA) is an autoimmune disease characterized by chronic inflammation of the synovial joints, pannus formation, progressive bone erosion, and joint destruction. Patients typically present joint swelling and tenderness, which can progress to serious disability, severely affecting the quality of the patient’s physical and mental life (1). RA affects approximately 1% of the world’s population and disproportionally affects female population (2). RA development is a continuous, progressive, systemic pathology process and multiple autoantibodies, including rheumatoid factor (RF) and anti-citrullinated protein antibodies (ACPA), are detectable in serum before the onset or in the early stage of RA (3–5). With the gradual interaction of various immnue and fibroblast cells and cytokines, synovial tissue gradually produces chronic inflammation accompanied by bone erosion and destruction, resulting in various clinical symptoms and injuries (3–5). Further, a number of organ systems can be damaged by the systemic inflammation, such as cardiac tissue, vascular system, kidneys, lung tissue, and the nervous system (6–8).

Ideally, chronic disease management approaches include preventive strategies. Modern medicine utilizes therapies that target the disease mechanism, the so-called “precision individualized diagnosis and treatment.” Discerning the specific environmental, cellular and molecular mechanisms suitable for early intervention is challenging given the complexity of etiological factors that give rise to RA. However, there is evidence that clinical monitoring of RA symptomology can improve the physical and mental health of patients (9, 10).

The pathogenesis of RA is thought to be involved with the interaction of genetic, epigenetics (11), environmental, metabolic, immune, and microbial factors. The relationship between genetic, epigenetic, metabolic, and microbial factors and RA has been widely reviewed (12–14). The progression of the disease is also influenced by the crosstalk among a variety of immune cells, such as T cells, B cells, monocytes, macrophages, neutrophils, mast cells, dendritic cells, T-reg cells, and fibroblast-like synoviocytes (FLS). These immune cells show plasticity in the disease microenvironment and heterogeneity in their roles depending on the context of the disease. Importantly, the mechanisms for immune cell-mediated synovial inflammation and cartilage destruction may not be active in all patients, and the extent of these effects varies from patient to patient and across the disease stages. Patients may show resistance (poor to no response) to one treatment and significant recovery with another. The considerable degree of clinical heterogeneity in RA affects the accuracy of a patient’s prognosis. Therefore, our most urgent challenge is to evaluate the heterogeneity inherent in the pathophysiology of RA and to identify the mechanisms of action in important cell subsets. Here, we summarize and comment on a variety of heterogeneous features to inform the development of precision and personalized medicine to RA which includes genetic variation, the various expression patterns in the synovium, and the heterogeneity of RA relevant cells, such as FLS, monocytes, macrophages, and mast cells.

## Genetic Heterogeneity in Rheumatoid Arthritis: Susceptibility and Clinical Implications

RA is a heritable autoimmnune disease mediated by genetic (15), epigenetic (11, 16), genetic-epigenetic (17) and genetic-environment (18) interactions while treatment usually take epigenetics and immnue factors as therapeutic targets (19). These inherited factors influence the heterogeneity of disease progression and determine the underlying set of cellular and molecular mechanisms. The role of heritability has been dissected in linkage and association studies, revealing the molecular variation underlying RA risk variability. The most evident aspects include (i) class II *HLA* genes, primarily *HLA-DRB1*, segregate variants that elevate RA risk 2–3-fold through aberrant antigen presentation (20–22); (ii) the 620W allele of the *protein tyrosine phosphatase nonreceptor 22* (*PTPN22)* gene, which generates various effects across numerous immune cell types and confers elevated risk of autoimmunity (15, 23); (iii) chemokine receptor genes, including *C-C chemokine receptor 6 (CCR6)* (15, 24, 25); (iv) *peptidyl arginine deiminase type IV (PADI4)*, which encodes peptidylarginine deiminase type 4 (involved in protein citrullination) (26, 27); (v) the transcription factor-encoding gene *signal transducer and activator of transcription 4 protein (STAT4)* (15, 25), (vi) *cytotoxic T-lymphocyte antigen 4 (CTLA4)*, encoding for the cytotoxic T-cell associated protein (25, 28), and (vii) the B-cell cell surface receptor gene, *CD40* (29, 30). Although these genetic factors predispose individuals to RA, patients exhibit a wide variety of combinations of these variants. This genetic variation across patients is considered to partially drive the heterogeneity in RA pathophysiology, clinical presentation, and response to therapies (31) (See **Table 1**).

**Table 1:** Main molecular variation underlying the RA risk variability.

Items	Relationship with RA	Ref.
class II HLA genes	Increases the risk of RA by enhancing the ability of cell antigen presentation, such as HLA-DRB1 haplotypes	(20–22)
the 620W allele at PTPN22	Some regional differences have been observed; increases the risk of RA by regulating B and T cell-mediated autoimmune responses	(15, 23)
CCR6	Acts as a potential pathogenic gene	(15, 24, 25)
STAT4	Acts as a potential pathogenic gene	(15, 25)
PADI4	Related to the citrullination of arginine residues in RA	(26, 27)
CTLA4	Acts as a potential pathogenic gene; encodes for the cytotoxic T-cell associated protein	(25, 28)
CD40	The SNP in CD40 affects the immune system in RA by regulating the expression of CD40	(29, 30)
rs7607479	Protects RA joints with positive autoantibodies by regulating the expression of SPAG16 and MMP in RA synovium and FLS	(32)
rs2900180	Possibly related to bone and joint erosion in RA	(33)
rs2833522	Contains H3K4me3 histone markers, transcription factors, and long non-coding RNA, which are related to the degree of bone destruction in ACPA-negative RA patients	(34)
rs6427528	Associated with changes in disease activity score after treatment with the anti-TNF-α drug (etanercept) by regulating CD84	(35)
rs7195994	Associated with the response to anti-TNF-α therapy (infliximab)	(36)

RA genetic risk factors, which underlie molecular and cellular perturbations, exhibit substantial differences across ethnic backgrounds. Indeed, this is a feature of many autoimmune diseases, such as multiple sclerosis, systemic lupus erythematosus, and type 1 diabetes. There are variations in the molecular pathophysiology of RA that vary across ancestral genetic backgrounds. For example, the 620W allele of the *PTPN22* gene displays a remarkable frequency gradient across Europe and is largely absent outside the continent (37). Therefore, the impact of the allele on critically important autoimmune mechanisms (e.g., T-cell activation and B cell pruning) has this geographical variation. The effects of *PADI4* on RA susceptibility, which may be driven by citrullination of arginine residues and/or leukocyte development, mostly affects East Asian patents. As clinical treatments progress toward more individualized approaches, a thorough understanding of these molecular effects will aid both therapeutic development and clinical implementation by medical practitioners. Seropositive RA (ACPA-positive and/or RF-positive) carries a different genetic susceptibility profile compared to seronegative RA. This fundamental difference in RA subtypes may be partly due to differences in genetic factors within the patient population. For example, *HLA-DRB1* haplotypes and *PTPN22* R620W are primarily correlated with seropositive disease (23, 38), whereas other susceptibility variants are similar between seropositive and seronegative diseases.

The set of RA susceptibility variants is likely to play a role in disease severity. A genome-wide study of 384 autoantibody-positive RA patients showed that the single nucleotide polymorphism (SNP) at position 2q34 of sperm-associated antigen 16 (*SPAG16*) in monocytes, rs7607479, has a protective effect on joints. This SNP primarily regulates the secretion of matrix metalloproteinases (MMPs), the expression of SPAG16 protein, and mRNA in the RA synovium and FLS (32). Baseline ACPA positivity is associated with increased radiological damage, and the genetic polymorphism rs2900180 in the TRAF1/C5 locus is associated with aggressive disease (33). In addition, a genome-wide study of 262 ACPA-negative RA patients showed that 33 SNPs were associated with joint destruction. Among them, the rs2833522 region contains H3K4me3 histone markers, transcription factors, and long non-coding RNAs, which are related to the degree of bone destruction in ACPA-negative RA patients (34). A modern and more accurate method of predicting the impact of SNPs on RA-related radiological characteristics involves the combination of genome-wide association study (GWAS) with bioinformatic analysis and repeated machine learning (39).

Although the treatment for RA has advanced considerably over the past few decades, nonsteroidal anti-inflammatory drugs (NSAIDs), DMARDs, corticosteroids, and biologics have shown an unequally efficacy cross some patients (40). This variation in the response to therapy also has a genetic component. Some studies have explored the effect of TNF-α gene polymorphism on anti-TNF-a therapy. Among them, single nucleotide polymorphisms (SNPs)-308 G/G, -857 C/T, +489 GG and GA, HLA-DRB1-encoding SE (allele *0404 and allele *0101) is beneficial to etanercept, while SNP-308 A/A and TNFR1A AA has the opposite effect (41, 42). Employing the dialogue on reverse engineering assessment and methods (DREAM), a Gaussian regression model was created to predict the changes in the DAS28 scores of patients with RA receiving tumor necrosis factor-α (TNF-α) inhibitor therapy, by using the expression patterns of genes involved in insulin secretion (*PDZD2*), immune response (*CD84*), and eicosanoid synthesis (*PLA2G4A*). Complex geographical variation has caused obstacles in the modeling of genetic markers. However, using extensive genetic subtype modeling, it was found that specific genetic markers were shared by particular populations and their identification could improve the prediction of efficacy of anti-TNF-α therapeutics (43). The SNP at position 1q23 (rs6427528) is associated with changes in RA’s disease activity scores in patients treated with etanercept, an anti-TNF-α drug. The SNP may disrupt transcription factor binding site motifs of the 3’ UTR of an immune-related gene, *CD84* (an immune-related gene), and the allele related to a better response to etanercept was linked to higher gene expression levels of *CD84* in peripheral blood mononuclear cells (35). In addition, studies have shown that the PTPRC SNP (rs10919563) was associated with an excellent response to anti-TNF-α therapy in patients with RA (36), and the rs7195994 variant at the FTO locus has been related to a clinical response with infliximab (44). In the genome-wide data for the RA population’s response to methotrexate (MTX), ten new risk sites were associated with poor MTX response. The genes that have been verified include *GHFR*, *FGPS*, and *TYMS* (all three related to purine and nucleotide metabolism) and *ENOSF1* (which interacts with *TYMS* at the transcriptional level) (45).

## Heterogeneous Expression Pattern of Synovial Tissue in Rheumatoid Arthritis

The structure of healthy synovial tissues has been well described (46). Understanding the difference between healthy and abnormal synovial tissue structure is essential for exploring the heterogeneity of RA. Healthy synovium consists of two parts: (i) the intima layer composed of macrophage-like synoviocytes (MLSs) and FLSs and (ii) the vascularized sublining layer comprising fibroblasts, macrophages, and the collagenous extracellular matrix (46, 47). Maintaining a healthy synovium is essential for optimal joint function and structure. By supplying nutrients for chondrocytes and preserving various cellular components of the synovial fluid, the synovium also plays a vital role in the homeostasis of the joint microenvironment.

The FLSs (unlike synovial macrophages) in the synovial intima express CD55; this expression can be used to discriminate FLSs from synovial macrophages (47, 48). Intimal FLSs also produce hyaluronic acid to inhibit adhesion and promote lubrication of joints, and the rate of secretion of hyaluronic acid depends on the mechanical stimulation of FLS. In addition, intimal FLSs coregulate immune responses with macrophages derived from monocytes (46).

In RA, the number of cells in the synovial intima substantially increases, primarily because of the abnormal proliferation of FLS. Macrophages flow into the vascular compartment, induced by a variety of cytokines and chemokines, and release various pro-inflammatory factors. These immune cells cooperate with FLSs and B cells to facilitate inflammation (49, 50). The salient feature of RA synovial tissue is the formation of a pannus (a source of matrix-degrading enzymes), which exacerbates cartilage erosion, inflammation, and joint destruction (51); Multiple histological studies have shown that the pannus is primarily composed of fibroblasts, macrophages, multinucleated neutrophils, and mesenchymal cells, though the roles of these cells in RA vary (52–60). With synovial biopsy technology, researchers can evaluate the cartilage-pannus junction (CPJ) and non-CPJ sites in human synovial tissue to identify the mechanisms and characteristics of cartilage and bone erosion in RA. Cell populations vary between the CPJ and non-CPJ. The most common cell populations in the CPJ include macrophages, FLSs, osteoclasts, chondrocytes, and mast cells. In the non-CPJ, the sublining layer has T cells, B cells, differentiated plasma cells, antigen-presenting cells, natural killer cells, and macrophages, and is characterized by synovial neovascularization. The dominant cell populations are FLSs and macrophages (61). Pannocytes, a unique type of rhombohedral cells, exist in the CPJ site and express high levels of transcriptional proto-oncogenes *c*-*FOS*, c-*MYC*, and c-*JUN* and express MMP-1, cathepsin B, and cathepsin L. Such expression may represent the early stage of mesenchymal cell differentiation; however, the role of pannocytes in bone destruction in RA remains to be elucidated (62, 63).

Some studies have described four modes of synovial histology in RA, including lymphoid, myeloid, pauci-immune, and fibroid variants (64, 65). All four models have unique gene expression characteristics. Based on such characteristics, the corresponding biomarkers should be identified to have reference values for different therapies. Lymphoid variants express high levels of genes related to the activation and differentiation of lymphoid B cells and lymphoid T cells, immunoglobulin production, antigen presentation, and cytokine signaling (Jak/STAT, interleukin [IL]-17), including *CD19*, *CD20*, *XBP-1*, *CD38*, *CXCL13*, and down-regulate the genes expression of *TGF-α*, and down-regulate the genes expression related to Wnt signaling, mesenchymal cell proliferation, proteolysis, cell transport, and ribosome metabolic processing (64). The presence of autoimmune B cell antigens and other lymphoid factors may promote the progression of synovitis and lead to a poor response to anti-TNF-α therapy (64). In addition, accumulation of cell aggregates reflects the proliferation of B and T cells in the lymphoid synovium. The existence of a large number of such aggregates may be one of the reasons for poor responses to anti-TNF-α therapy in RA (66).

Myeloid variants express high levels of genes related to chemotaxis, *TNFα* and *IL-1β* production, Toll-like receptor and nucleotide-binding oligomerization domain (NOD)-like receptor signaling, Fcγ-receptor-meditated phagocytosis, and proliferation of mononuclear cells. They highly activate nuclear factor kappa-light-chain enhancer of activated B cells (NF-κB) pathway genes, including *TNFα*, *IL-1β*, *IL-1Rα*, *ICAM1*, and *MyD88*, *the inflammatory chemokines CC-chemokine ligand (CCL)-2* and *IL-8*, granulocyte and inflammatory macrophage lineage genes such as *S100A12*, *CD14* and *OSCAR*. In contrast, they downregulate the expression of genes related to transcription and splicing. The myeloid synovial tissue has fewer cell aggregates (primarily containing pro-inflammatory M1 mononuclear macrophages), and it highly expresses a variety of inflammatory genes; therefore, such tissues are more responsive to anti-TNF-α therapy (64). Studies have shown that RA synovium across joints that express high levels of inflammatory genes, have high levels of inflammation (i.e., high DAS28 score, high CRP level, high erythrocyte sedimentation rate, high platelet count, and a shorter course of disease) (28);

The pauci-immune synovium is primarily enriched in genes involved in the process of inflammation and wound healing. It shows gene expression patterns related to several other phenotypes, and expresses high levels of IL-6, the IL-6 receptor (IL-6R) components *IL-6ST/gp130 and STAT3*. Such expression patterns are consistent with previous studies on the biological role of IL-6 in RA. The pauci-immune synovium is primarily characterized by low levels of inflammation and accumulation of anti-inflammatory M2 monocytes/macrophages, which increases the potential for a poor response to anti-TNF-α therapy and B cell depletion therapy (64, 67–70).

Fibroid variants are primarily enriched in genes involved in the regulation of FLSs and osteoclasts/osteoblasts, such as transforming growth factor (TGF)-β signaling, bone morphogenetic protein (BMP) signaling together with associated Sma Mothers Against Decapentaplegic (SMAD) binding, as well as endocytosis and cell projection process-related genes. They express high levels of Wnt pathway genes while significantly downregulating multiple immune-system processes (associated with B cells, immunoglobulins, myeloid cells), the innate immune response (including NOD-like receptor signaling), and chemotactic processes. The expression levels of angiogenesis-related genes in fibroid variant synovium is high, which is correlated with reduced response to anti-TNF-α therapy (64).

Importantly, RA seropositive patients are limited to lymphoid, myeloid, and pauci-immune types, and both soluble intercellular adhesion molecule-1 (sICAM-1) and CXCL13 levels are elevated in these patients. The sICAM-1 and CXCL13 levels can represent the two synovial phenotypes of myeloid lymphoid cells, respectively, and can be used as a biomarker for predicting anti-TNF-α and anti-IL-6R therapies. Synovial migration of inflammatory cells can be induced by sICAM1 binding to receptors of neutrophils and monocytes in response to TNF-α stimulation (64, 71). CXCL13 is a B-cell-specific chemokine that plays a key role in secondary lymphoid tissues and germinal center tissues (72). IL-6/IL-6R and lymphoid B cell-driven synovitis are highly correlated (a consistent result in multiple studies), but in myeloid synovial tissue dominated by activated monocytes, anti-TNF-α therapy is most effective. A major limitation is the necessity for multiple biomarkers to accurately apply RA therapy. Predicting the efficacy of different therapies for RA based on the heterogeneity of synovial tissue is a promising direction for future research (64, 71, 73, 74).

## Mast Cells, a Novel Target for Treating Rheumatoid Arthritis, Which Contributes to Therapeutic Heterogeneity

Mast cells (MCs) consist primarily of two subtypes, tryptase-positive (MC_T_) cells and tryptase/chymase double-positive (MC_TC_) cells, and play a role in RA by promoting inflammation, osteoclast differentiation, and angiogenesis (75). Tetlow et al. studied the activation and degranulation of MCs at the CPJ. Levels of stromelysin-1, TNF-α, IL-1β, extracellular MC tryptase, and matrix degradation mediators at the CPJ site are all significantly elevated, suggesting that MC degranulation is related to local matrix degradation and inflammation (76). In RA synovial tissue, the number and proportion of MC subpopulations are characterized by significant modifications. In normal synovium, mast cells account for approximately 5% of synovial cells and primarily contain MC_TC_ cell subpopulations. Gotis-Graham et al. found that the number of synovial MC_T_ cells in early RA patients increased to three times the number of MC_TC_ cells and this ratio strongly correlated with histological inflammation (77). The researchers found that RA patients in the active stage of the disease have a higher number of MCs than patients with end-stage RA (78). In addition, MCs can promote inflammation by releasing various chemokines and pro-inflammatory factors in cooperation with other cells. Mast cells mediate the release of IL-6, IL-8, TNF-α, histamine, heparin, monocyte chemoattractant protein-1 (MCP-1), MIP-1α, and RANTES, and they stimulate monocytes/macrophages to produce/release IL-1 family members and TNF-α, which exacerbate inflammation (79–82). MCs can also participate in bone destruction by promoting the release of proteases, histamine, TNF-α, IL-6, IL-11, IFN-γ, RANKL, and other mediators. MCs increase the expression level of TNF-α, IL-1β, IL-6, IL-17, RANKL, and MMP-9 genes in response to IL-33 stimulation and stimulate human CD14+ monocytes to differentiate into TRAP-positive osteoclasts (83). Shin et al. found that secretion of tryptase β by MCs was abundant in experimental models of arthritic mice, and the tryptase β/heparin complex promoted FLS to express inflammation-mediating neutrophil chemokines CXCL1/KC, CXCL5/LIX, and CXCL8/IL-8; additionally, tryptase β-deficient mice showed lower disease activity and bone destruction (84). Another study found that experimental models of arthritic mice lacking mouse MC protease-4 had lower clinical scores, as well as lower levels of cartilage destruction and pannus formation (85). Studies have shown that histamine released by MCs can stimulate the expression of the histamine H4 receptor and RANKL in monocytes and induce osteoclast differentiation (86). Vascular endothelial growth factor (VEGF), tryptase, and fibroblast growth factor-2 (FGF-2) released by MCs can stimulate FLS proliferation and promote the formation of pannus (87). Studies have also found that CXCR3 is strongly expressed on the surface of mast cells in synovial tissues, leading to increased recruitment of MCs and increased levels of chemokines such as CXCL9 and CXCL10, exacerbating RA (88).

## Monocyte Heterogeneity and Plasticity in Rheumatoid Arthritis Pathogenesis

The origin and typing of monocytes have been extensively studied across multiple species, including humans, mice, pigs, monkeys, and horses (89). Monocytes were first defined in a classic study conducted by Ehrlich et al. in the 20^th^ century (90). Monocytes are circulating blood cells that develop in human bone marrow (BM) from the common myeloid progenitor (CMP). Together with macrophages and dendritic cells, they are members of the mononuclear phagocyte system (91, 92). The widely used monocyte subpopulation classification standard is based on the expression of CD14 and CD16. The monocyte population is primarily divided into three subpopulations: the classic monocyte population (CD14++), which accounts for approximately 80% of the blood mononuclear cells in healthy individuals, the intermediate monocyte population (CD14++CD16++), and the non-classical monocyte population (CD14+CD16++). The latter two types of CD16-expressing monocytes account for a relatively small proportion in healthy individuals, but their number can increase in different pathological conditions (93). Changes in monocyte subpopulations can be used as biomarkers of RA disease activity and to assess the clinical response. Studies have reported irregularities of monocyte subsets in patients with RA. The proportion of classical monocytes in patients with early RA is relatively low, and the proportions of intermediate and non-classical monocytes are relatively high (94). Similarly, Tsukamoto et al. found that the proportion of intermediate monocyte subpopulation was positively correlated with RA disease activity and negatively correlated with the prevalence of the classical monocyte subpopulation; intermediate monocytes express CD16 under the stimulation of IL-10 (95). In addition, studies have found that classic monocytes and intermediate monocyte subpopulations can predict the clinical response to MTX (96). Increased expression levels of CD16 on CD14++ monocytes in RA patients leads to an intensified response of IgG-containing immune complexes (IC) and the excessive production of pro-inflammatory factors. This change may cause a lack of responsiveness to MTX therapy (97).

Monocytes display phenotypic heterogeneity and plasticity, expressing a variety of cell receptors and secreting various cytokines, including CD14, CD16, HLA-DR, Toll-like receptor, B1 integrins, B2 integrins, a proliferation-inducing ligand (APRIL), CCR2, CX3CR1, siglec-1, and interleukin family cytokines (e.g., IL-18, IL-1β, IL-6, IL-1, IL-32, IL-33, IL-10, and IL-11); depending on the microenvironmental stimuli, they can differentiate into macrophages and dendritic cells (98). Further clarification of the different mechanisms of expressed receptors and secreted cytokines can inform targeted clinical treatment plans. In response to LPS stimulation and binding to the LPS-binding protein in the plasma, the toll-like receptor (TLR)-4 binds to CD14 on the cell membrane, inducing the release of different mediators, including pro-inflammatory chemokines (e.g., IP10) and cytokines (e.g., TNF-α, IL-6, and IL-1) (99, 100). CD16 (Fc gamma RIII) is another receptor for the lgG Fc fragment expressed in the monocyte population (101). Compared with that in a control group, the number of monocytes expressing CD16+ increased sharply in RA patients (102). McGarry et al. found that CD14+ monocytes displayed mitochondrial respiration, biogenesis, enhanced glucose consumption and mitochondrial morphological changes, and they enhanced gene expression of key glycolytic enzymes, such as *HIFIα*, *HK2*, and *PEKFB*. Blocking of STAT3 inhibits this forced glycolytic flux along with inflammation and may represent a potential therapeutic approach for preclinical RA (103). Belge et al. found that, compared with classic monocytes, CD14++CD16++ monocytes expressed higher levels of HLA-DR antigens and can produce higher levels of TNF-α. The mechanism involves an interaction of CD14 and Pam3Cys with TLR2 (104, 105). Yoon et al. showed that in the presence of TGF-β, IFN-γ induces the expression of HLA-DR and CD80 and CD276 in synovial intermediate monocyte subpopulations, which act as signal one and signal two, respectively, to induce CD4+ T cell polarization to Th1 and Th17 cells, which secrete more IFN-γ and IL-17. CD16+ monocytes can also promote the release of TGF-β, which promotes synovial inflammation through positive feedback (106). Iwahashi et al. showed that the human mitochondrial protein HSP60, which is widely expressed in RA synovium, can induce CD16+ monocytes to produce TNF-α, possibly through cascade activation of MyD88-NF-κB-mitogen-activated protein kinase (MAPK) (107). The classic and intermediate monocyte subpopulations in the synovium and blood of RA patients express TLR2 in large quantities, and all three monocyte subpopulations express TLR9; and the subpopulation of classical monocytes is triggered produces pro-inflammatory cytokines in response to TLR2 and TLR9 agonist stimulation (108). FLS expresses TLR1–6 (TLR3 and TLR4 are highly expressed), which may be involved in the activation of pathways related to inflammation and joint destruction in early RA (109). Thwaites et al. showed that the activation of monocyte surface receptors TLR1 and TLR2 in patients with RA mediated the increase in the levels of IL-6 and TNF-α, and the activation of TLR5 mediated the increase in the levels of IL-6 and IL-10. IL-6 induced by TLR1/2 is related to the DAS28 score, a commonly used score to measure disease activity in RA (110). Alpha-enolase (ENO-1) can be produced by FLSs, promoting the proliferation and survival of the cells and can combine with TLR4 on monocytes to produce pro-inflammatory factors and chemokines, such as TNF-α, IL-1β, IL-6, CCL3, IL-8, and CXCL1 (111, 112). In addition, the levels of surface expression on the monocyte subpopulations for transmembrane TNF (tmTNF) are high in all RA patients. Anti-TNF antibodies can act as ligands, binding to tmTNF and mediating reverse signal transmission (113). Anti-TNF binding to tmTNF can also exert anti-inflammatory effects by inducing the release of the decoy receptors sTNFR1, sIL-1R1, and sIL-1R2 and increasing the production of IL-10. In addition, patients whose monocytes express TNFR1 have a lower disease activity score, which may be due to inhibited inflammation caused by sTNFR1 neutralizing TNF or through the apoptosis of pro-inflammatory monocyte subpopulations by the activation of the TNFR1 exogenous death receptor pathway (114). Monocytes can act as antigen-presenting cells to activate lymphoid T cells. Monocyte-derived dendritic cells (Mo-DCs) can be produced under IL-4/granulocyte-macrophage colony-stimulating factor (GM-CSF) conditions to induce CD4+ T cell polarization to Th17 and Treg cells and increase the release of IL-6 and IL-23 (115). Jongbloed et al. found that the extent of two heterogeneous subsets of dendritic cells, myeloid DCs (mDCs) and plasmacytoid DCs (pDCs), were significantly increased in the synovial fluid of patients with RA (116), pDCs can activate B cells to differentiate into plasma cells and secrete antibodies in T cell-dependent and T cell-independent manners. The number of pDCs showed a significant positive correlation with the serum level of ACPA (117–120). Compared with normal monocytes, monocytes in RA also express higher levels of various adhesion proteins, including fibronectin and the B2 integrin complex CD11b, which promote adhesion and migration (121). The monocytes in RA express proliferation-inducing ligands on the cell surface and release soluble forms, which are significantly related to disease activity. B cell activating factor (BAFF) combined with APRIL sustains the survival of autoimmune B cells and promotes an autoimmune response (122). By exploring the changes in serum APRIL in anti-CCP positive and negative patients treated with MTX and hydroxychloroquine for 6 months, it was found that the serum concentrations of APRIL increased compared with that of the control group, and they were positively correlated with disease activity, joint swelling, visual analog score, and a simplified disease activity index. In addition, the levels of APRIL in anti-CCP-positive RA patients were significantly lower than that in anti-CCP-negative RA patients after treatment. APRIL is highly expressed on the surface of all circulating monocyte subsets in patients with RA and is related to disease activity (123, 124). A high concentration of APRIL was detected in the synovial homogenate of rats with adjuvant-induced arthritis (AA); APRIL stimulated FLS proliferation, migration, and secretion of pro-inflammatory factors (125). Siglec-1 levels in RA peripheral blood mononuclear cells and monocyte subpopulations were significantly increased in response to TNF-α, IFN-γ, and type II collagen stimulation and was positively correlated with clinical disease indicators (e.g., DAS28, ESR, CRP, and IgM-RF) (126). Monocytes, macrophages, and FLSs in RA increase the expression levels of protease-activated receptor-2 (PAR2); they promote the production of IL-6 and TNF-α and enable the proliferation and invasion of FLSs (127, 128).

## Macrophage Heterogeneity in the Personalized Treatment of Rheumatoid Arthritis

There are primarily two macrophage subsets in the peritoneal cavity of the arthritic mice, namely large peritoneal macrophages (LPMs) and small peritoneal macrophages (SPMs). LPMs are derived from fetal liver macrophages, and their major markers are CD11b^high^, CD11c^low^, F4/80^high^, CD209^-^, GATA-6^+^, MHC II^low/-^, CD62L^-^,and TIM4^+^. SPMs develop from bone marrow-derived monocytes, and their major markers are mainly CD11b^low^, CD11c^-^, F4/80^low^, CD209^+^, GATA-6^+^, MHC II ^high^, CD62L^+^, and TIM4^-^ (129–132). Two subsets of synovial macrophages were observed: F4/80^+^CD11b^-^ (releasing anti-inflammatory mediators such as IL-4 and IL-10) and F4/80^-^CD11b^+^ (bone marrow-derived; releasing pro-inflammatory mediators such as IL-1 β and TNF- α) (133). In addition, it has been found that CX3CR1+ tissue-resident macrophages subsets can isolate joints and inhibit the inflammation by forming tight junction barriers with membrane-like structures (134).

Many macrophages can be found in the synovial tissue in the active stage of RA, and the number of macrophages after clinical remission is reduced, indicating that macrophages are and the number of macrophages decrease after clinical remission, indicating that macrophages are highly plastic and can respond to microenvironmental stimuli (e.g., *via* polarization). Many studies have shown that changes in macrophage prevalence in synovial tissue can predict the effect of treatment. The changes in the number of macrophages in the sublining layer are significantly different among good responders, moderate responders, and non-responders after clinical treatment; these changes are strongly related to changes in DAS28 scores, with excellent sensitivity (135, 136). Activated macrophages recruit immune cells and FLSs by producing IL-1β, IL-6, TNF-α, IL-12, and other cytokines to promote inflammation, while chronic inflammation and cytokines secreted by other cells assist in the activation, polarization, and apoptosis of macrophages (132). The expression levels of macrophages at the CPJ and non-CPJ sites were significantly different. Youssef et al. discovered the RA synovial expression pattern of myeloid related proteins (MRPs), which are macrophage activation markers. Macrophages expressed MRP8, MRP14, and MRP8/14 dimers in the lining layer of the CPJ only in the active stage of the disease, indicating that the macrophages were activated and polarized at the cartilage destruction site in RA (137).

**Figure 1:**
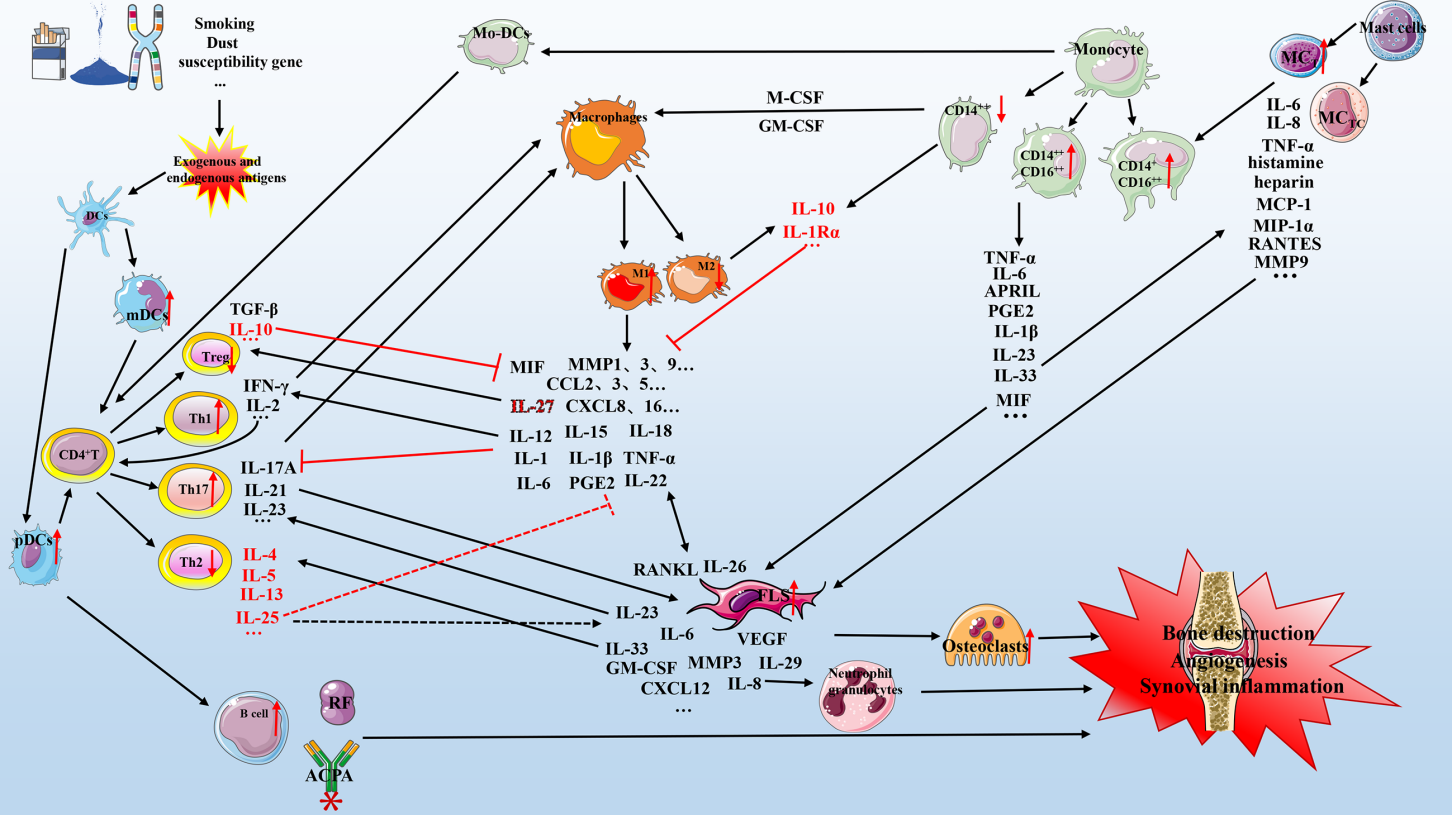
Mechanism of cell-cytokine interaction in RA. Various factors (e.g., smoking, dust, genetic factors, and microorganisms) lead to the production of exogenous and endogenous antigens. Antigen-presenting cells (primarily dendritic cells) present exogenous and endogenous antigens to CD4+ T cells that differentiate into T cells with different functions, including Th1, Th2, and Th17. These cells cooperate with mast cells, macrophages, and monocytes to secrete multiple pro-inflammatory mediators that act on FLSs and osteoclasts, which in turn can secrete various biological mediators to aggravate the circulation. The anti-inflammatory mechanism (red represents anti-inflammatory mediators) is active but insufficient to inhibit the pro-inflammatory process (black represents pro-inflammatory mediators). Briefly, the interaction of various cell subgroups and cell mediators forms a complex network that promotes the development of RA, including bone destruction, angiogenesis, and synovial inflammation. DC, dendritic cells; IL, interleukin; TNF-α, tumor necrosis factor-α; mDCs, myeloid DCs; pDCs, plasmacytoid DCs; GM-CSF, granulocyte-macrophage colony-stimulating factor; M-CSF, macrophage colony-stimulating factor; CXCL, chemokine CXC ligand; CCL, CC-chemokine ligand; MMP, matrix metalloproteinase; MCP-1, monocyte chemoattractant protein-1; PGE2, prostaglandin E2; APRIL, a proliferation-inducing ligand; RF, rheumatoid factor; ACPA, anti-citrullinated protein antibody; FLS, fibroblast-like synoviocyte; TGF, transforming growth factor; VEGF, vascular endothelial growth factor; MC, mast cell.

The concept of macrophage polarization helps distinguish the biological functions of macrophages in different states and their characteristics in the pathophysiology of disease. Studies have tested the expression of macrophage populations in multiple locations associated with RA. The synovial lining layer macrophages primarily display an IL-10 polarized-like phenotype, indicating the dominance of M2 macrophages. The macrophages in the synovial sublining layer co-express M1 and M2 markers, indicating a more heterogeneous phenotype (138). Palacios et al. found that macrophages in RA synovial fluid and synovium highly express pro-inflammatory polarizing genes, including *INHBA*, *MMP12*, *EGLN3*, and *CCR2*, and show low levels of expression of anti-inflammatory genes, including *IGF1*, *HTR2B*, *FOLR2*, *SERPINB2*, and *CD36* (139). ACPA can induce the activity of interferon regulatory factor-5 (IRF5) to promote polarization toward M1 macrophages and increase the ratio of M1 to M2 cells (140). M1 macrophages were observed to be predominant among peritoneal macrophages in collagen-induced arthritis (CIA) mice; this finding is consistent with the previous result (141). Therefore, there is an imbalance between the number of M1 and M2 macrophage subpopulations in RA. M1 is predominant and promotes inflammation. Although the polarization mechanisms of M1 and M2 have not been fully elucidated, both M1 and M2 macrophages secrete different cytokines and mediators that cooperate with other cells to affect disease progression by responding to corresponding stimuli (**Figure 1**).

## Interaction of Cell Subsets and Cytokines in Rheumatoid Arthritis

The RA pathogenesis is complex and involves the interaction of various innate and adaptive immune cells, which secrete various chemokines, inflammatory mediators, pro-inflammatory factors, anti-inflammatory factors, and other substances that act on the patient’s synovial tissue and joints. At the initial stage of RA, DCs present exogenous and endogenous antigens to CD4+ T cells by expressing HLA-DR molecules. CD4+ T cells can differentiate into Th1, Th2, and Th17 cells secreting different cytokines, which have different functions in RA. The levels of IL-17, IL-21, and IL23 secreted by Th17 are significantly increased in the synovial fluid of RA patients and are positively correlated with DAS28, CRP, and ACPA. IL17A has a variety of functions. It can stimulate FLS and macrophages to produce VEGF, IL-1, IL-6, TNF-α, and prostaglandin E-2 (PGE-2), and can promote RANKL expression to stimulate synovial inflammation, angiogenesis, and osteoclast formation. In addition, IL-17, IL-1, and TNF-α upregulate the levels of IL-6, MMP, and type I collagen and participate in bone destruction (142). Different subsets of monocytes can secrete various cytokines, including IL-1β, IL-6, IL-18, IL-32, TNF-α, IL-33, IL-23, IL-10, and IL-1Rα and express various cell receptors that cooperate with other cells in RA in different ways. Monocytes can act as antigen-presenting cells to activate CD4+ T cells to differentiate into Th1 and Th17 cells. IL-18 is a key factor in Th1 response and cooperates with IL-12 and IL-15 to induce IFN-γ production in synovial tissue and promotes the production of pro-inflammatory factors, such as TNF-α and IL-1, in an IFN-γ-independent manner. IL-10 can inhibit the production of TNF-α and IFN-γ, and IL-1β and TNF-α promote IL-18 release by FLSs to strengthen this effect (143–145). IL-32 is highly expressed in RA synovial tissue and can induce PGE2 release from mouse macrophages and human blood monocytes. Synovial IL-32 staining was positively correlated with the indices of synovial inflammation, TNF-α, IL-1β, and IL-18 (146). Monocytes exposed to the synovial fluid of RA express lower levels of IL-10 and higher levels of IL-1Rα, which may result from the anti-inflammatory mechanism; however, this effect does not offset the rest of the pro-inflammatory process (147). IL-6 and IL-1β produced by monocytes can promote inflammation and increase adhesion (121). Monocytes and FLSs can also secrete IL-23 and IL-33. IL-23 is essential for Th17 cell differentiation and IL-17A production (148, 149). IL-33 can induce Th2 cell differentiation; stimulate the production of IL-4, IL-5, and IL-13; and promote inflammation by binding to ST2 receptors on MCs to release related cytokines (150–152). Th1, Th17, FLSs, and monocytes can secrete GM-CSF, induce polarization of macrophages into M1 phenotypes through IRF4 or IRF5, and promote the production of IL-6, IL-23, and CCL17 to induce inflammation (153). They can also secrete M-CSF, which induces macrophage polarization toward M2 type, thereby exerting an anti-inflammatory mechanism (154).

M1/M2 macrophages can secrete a variety of biological mediators, including IL-1, IL-1β, IL-6, IL-26, IL-27, IL-29, IFN-γ, IL-22, TNF-α, IL-12, IL-15, IL-18, IL-20, macrophage migration inhibitory factor (MIF), MMP-1, MMP-2, MMP-9, MMP-12, MMP-13, various CXCL chemokines, CCL2, CCL3, CCL5, ROS, IL-10, IL-1RII, and TGF-β. Serum IL-12 levels are positively correlated with the number of swollen joints, levels of RF, and other disease activity markers in patients with RA, and can be used as a predictor of disease activity (155). Vitamin D receptors expressed on the surface of macrophages. Also, vitamin D receptors are widely expressed on the surface of many immune cells, such as CD4+ T cell, CD8+ T cell, B cell, and DC cell. Vitamin D has roles in calcium metabolism, bone health, and anti-inflammatory, which has been widely reviewed in many diseases. It may be possible to partially alleviate various autoimmune responses by regulating maladjusted microbial populations in RA. The specific mechanism needs further study (156–160). IL-12 can also induce Th1 cell differentiation (161). In the presence of a large amount of TNF-α, Th1 cells release IFN-γ and IL-2, and IFN-γ further induces macrophages to polarize toward M1 phenotypes. IL-2 is an important regulator of T cell-dependent responses and plays a crucial role in T cell proliferation and survival (132, 162). The cytokine IL-22, produced by macrophages and Th22 cell subsets, is related to disease activity and promotes osteoclast production by inducing RANKL production in FLSs, while IL-25 produced by Th2 cells can inhibit this effect *via* the STAT3/P38/MAPK/IkBa pathway (163, 164). In addition, IL-25 attenuates the development of CIA by inhibiting the differentiation of CD4+ T cells into Th17 cells (165, 166). However, some studies have shown that IL-25 is involved in the aberrant proliferation of FLSs (167). M1 macrophages promote synovial inflammation by producing a large number of pro-inflammatory factors, including IL-1, IL-6, IL-1β, IL-12, and TNF-α (168). The level of IL-15 is increased in the serum and synovial fluid in the early stage of RA, and promotes the release of TNF-α and IL-17 to participate in T lymphatic activation and subsequent bone destruction. It can be used as an independent biomarker for the detection of early RA (169–172). IL-27 secreted by macrophages has dual anti-inflammatory and pro-inflammatory functions. It induces FLSs to release a variety of pro-inflammatory mediators, including IL-6, vascular cell adhesion protein 1 (VCAM1), CCL2, CXCL9, CXCL10, and MMP-1. On the other hand, IL-27 binding to the IL-27 receptor of T cells induces their differentiation into Treg cells that secrete IL-10, which inhibits Th17 differentiation as an anti-inflammatory mechanism (173–176). FLSs and macrophages produce IL-26 and IL-29, respectively. IL-26 can promote the polarization of M1 macrophages by activating the c-JUN/NF-κB/STAT1 signaling pathway and produce IL-6 and TNF-α pro-inflammatory factors (177). IL-29 is elevated in the serum of RA patients and participates in the chemotaxis of peripheral blood neutrophils, inhibits T follicular helper (Tfh) cell differentiation by reducing STAT3/BCL6 activity, and induces RANKL expression in FLSs through the MAPK pathway to participate in bone destruction. IL-29 upregulates the levels of TLRs in FLS to mediate the expression of IL-6 and IL-8, which promotes inflammation (178–182). IL-18 levels in the synovium and serum are related to RA disease activity. Inhibition of IL-18 can inhibit the secretion of pro-inflammatory factors such as IL-6, IL-18, TNF-α, and IFN-γ. Compared with those of other cytokines, IL-18 levels were significantly reduced after leflunomide treatment; therefore, the IL-18 serum levels can be used as a potential biomarker to measure the efficacy of leflunomide (183, 184). TNF-α inhibitor treatment can significantly reduce bone loss in patients with RA, which is related to decreased IL-20 and RANKL expression levels (185). MIF secreted by monocytes and macrophages promotes the secretion by Th1 and Th17 cells. Pro-inflammatory cytokines, including TNF-α, IFN-γ, IL-1β, IL-6, and IL-17A, induce the production of MMP-1, MMP-3, MMP-9, MMP-13, phospholipase A2, and cyclooxygenase-2, which induce FLS to produce VEGF, IL-8, and RANKL, and promote the differentiation of monocytes into osteoclasts, all of which mediate bone destruction in RA (186, 187). CXCL1–8 can combine with CXCR2 in ECs cells to participate in angiogenesis. Inhibition of CXCL4 may improve inflammation in CIA mice. The inhibition of CXCL8 reduces angiogenesis in RA, and the release of CXCL12 from FLS could inhibit the pro-angiogenic activity of other chemokines and VEFG by binding to CXCR4 or CXCR7 (188, 189). CCL2/3/4/5 and CXCL9/10 levels are increased in RA, of which CCL2/5 and CXCL10 promote osteoclastogenesis. The production of CCL2/MCP-1 from FLS and macrophages through the PI3K/ERK/JNK pathway could result in IL-17-induced monocyte migration from the blood to the synovium (190, 191). Briefly, the interaction of various cell subgroups in RA promotes inflammation and bone destruction. Although many anti-inflammatory mediators are secreted, the level of anti-inflammatory mediators is not sufficient to inhibit the process of chronic inflammation and bone destruction. About other existing cell subsets in autoimmune diseases, we intend to clarify the roles and their mechanisms for RA in the future, such as NK cells, neutrophils (192).

## Future Perspective: Biologics and Non-Biologic Drugs for Rheumatoid Arthritis

The common drugs used to treat RA include MTX and leflunomide, among others. Based on the heterogeneity of multiple cell subsets in different stages of RA, it becomes apparent why some patients with RA have poor or no response to treatment. Therefore, researchers have focused on developing biological therapies that target different cells or cytokines and have achieved some therapeutic success. When MTX and leflunomide show poor efficacy, the combined use of biologics may improve the response. There have been many notable reviews summarizing the progress of pharmacological research in this field (193, 194), which we have updated and briefly described in **Table 2**.

**Table 2:** RA-related biologic therapy and clinical trials.

Name	Target	ClinicalTrials.gov ID	Primary Outcome	Pharmacological Role	Ref.
Tocilizumab	IL-6R	NCT01951170	Change From Baseline in Genant-modified Total Sharp Score (mTSS) [Time Frame: From baseline to Week 24]	Inhibiting IL-6R	(195)
Rituximab	B cell	NCT02304354	DAS28 and T-lymphocyte count [Time Frame: up to week 48]	Primarily depletion of B cells, in addition to reduction of T cells and macrophages	(196)
		NCT01592292	(1) Mean Change From Baseline in DAS28 at Month 6 in Intention to Treat (ITT) Population [Time Frame: Baseline and Month 6]	Depletion of B cells	
			(2) Mean Change From Baseline in DAS28 at Month 6 in Standard Population Set (SPS) [Time Frame: Baseline and Month 6]		
		NCT02079532	Change From Baseline to Week 24 in DAS28 [Time Frame: Week 24]	Depletion of B cells	
		NCT01071798	DAS28 Score and HAQ Disability Index (HAQ-DI) [Time Frame: at baseline of each cycle and approximately 15 days, 6 weeks (only cycle 1), 12 weeks (3 months), 18 weeks (only cycle 1), and 24 weeks (6 months) after the start of the respective cycle]	Predicting biomarkers of clinical therapy	(197)
		NCT01126541	DAS28-CRP Area Under the Curve (AUC) at Week 104 [Time Frame: Week 104]	Predicting biomarkers of clinical therapy	(198–201)
		NCT00468546	Number of Participants With ACR 20 Response at Week 24 [Time Frame: Week 24]	Predicting biomarkers of clinical therapy	(202)
		NCT00147966	ACR 20 Response at Week 12 [Time Frame: 0 and 12 weeks]	Predicting biomarkers of clinical therapy	(74)
Infliximab	TNF-α	NCT00908089	Remission by ACR criteria [Time Frame: 2 years]	Inhibiting inflammation	(203)
Infliximab	TNF-α	NCT00213564	*NA*	Response factor prediction	
Infliximab	TNF-α、T cell、B cell、IL-6R	NCT01638715	Absolute Change in the Simplified Disease Activity Index (SDAI) [Time Frame: 24 Weeks]	Response factor prediction	
Tocilizumab					
Abatacept					
rituximab					
Adalimumab	TNF-α	NCT00195663	(1) Number of Participants Meeting ACR50 Response Criteria at Week 52 [Time Frame: Baseline and 52 Weeks]	Inhibiting inflammation	(204)
			(2) Change From Baseline in mTSS at Week 52 [Time Frame: Baseline and Week 52]		
Tabalumab	BAFF	NCT00689728	Percentage of Participants ACR 50 Response at Week 16 [Time Frame: 16 weeks]	Decreasing the number of autoimmune B cells	(205)
Spebrutinib(CC-292)	Bruton’s tyrosine kinase (BTK)	NCT01975610	ACR 20 Response [Time Frame: Week 4]	Inhibiting B cell proliferation and osteoclast production	
Tofacitinib	JAK1/3	NCT00976599	(1) Change From Baseline in Synovial Tissue Messenger Ribonucleic Acid (mRNA) Expression at Day 28 [Time Frame: Day -7 (Baseline), Day 28]	Inhibiting angiogenesis and reducing P-STAT1, P-STAT3	(206)
			(2) Change From Baseline in Protein Expression of TNF-α, IL-6, IL-17a, and IL-10 at Day 28 [Time Frame: Baseline (Day -7), Day 28] … etc. (82 items in total)		
Ustekinumab	IL-12/IL-23	NCT01645280	Percentage of Participants With ACR 20 Response at Week 28 [Time Frame: Week 28]	Inhibiting IL-12/IL-23	(207)
Anakinra	IL-1R	NCT00117091	Percentage of subjects continuing Kineret^®^ therapy at the end of the study (i.e., responders according to pre-defined response assessment criteria)	Inhibiting IL-1R	
KB003	GM-CSF	NCT00995449	This Study Was Initiated With a Safety run-in Period to Evaluate Acceptability of Repeat-dose Safety. [Time Frame: Weeks 14 and 30]	Inhibiting M1 macrophage polarization	
MOR103 (GSK3196165/Otilimab)	GM-CSF	NCT01023256	Percentage of Patients With Treatment-emergent or Serious Adverse Events [Time Frame: From the first dose through the 16-week visit]	Inhibiting M1 macrophage polarization	(208)
		NCT02799472	(1) Change From Baseline in Target Engagement Biomarkers- Soluble GM-CSF Complexed to GSK3196165 [Time Frame: Baseline and Weeks 1, 2, 4, 6, 8, 12, 12-Week follow-up (FU) (Week 22)]		
			(2) Change From Baseline in Predictive Biomarkers: 14-3-3 ETA Protein, S100 CBP A8 and A9 [Time Frame: Baseline and Weeks 1, 2, 4, 6, 8, 12, 12-Week FU (Week 22)] … etc., 19 items in total		
		NCT02504671	Percentage of Participants with DAS28-CRP Remission (DAS28 <2.6) at Week 24 [Time Frame: Week 24]		
		NCT03980483	Proportion of participants achieving ACR 20 at Week 12: superiority comparison with placebo [Time Frame: Week 12]		
		NCT03970837	Proportion of participants achieving 20% improvement in ACR20 at Week 12: superiority comparison with placebo [Time Frame: Week 12]		
		NCT04333147	(1) Incidence of adverse events (AEs), serious AEs (SAEs) and AEs of special interests (AESI) [Time Frame: Up to 4 years]		
			(2) Change from Baseline in platelet count, neutrophils, lymphocytes, monocytes, eosinophils, and basophils (Giga cells per liter [giga cells/L]) [Time Frame: Baseline (Day 1) and up to 4 years] … etc. (eight items in total)		
		NCT04134728	Proportion of participants achieving 20% improvement in ACR20 at Week 12 superiority comparison with placebo [Time Frame: Week 12]		
		NCT03028467	(1) Maximum Observed Concentration (C_max_) of GSK3196165 [Time Frame: Pre-dose on Days 1, 8, 15, 29, 57, and 71; anytime during visit on Days 3, 74, 85, 106, 127, and 155]		
			(2) AUC From Time Zero to the Time of the Last Quantifiable Concentration (AUC _[0-t]_), AUC From Time Zero Extrapolated to Infinity (AUC _[0-inf]_), AUC Over the Dosing Interval (AUC_tau_) of GSK3196165 [Time Frame: Pre-dose on Days 1, 8, 15, 29, 57, and 71; anytime during visit on Days 3, 74, 85, 106, 127, and 155]		
			(3) Time to Reach C_max_ (T_max_) and Terminal Half-life (t1/2) f GSK3196165 [Time Frame: Pre-dose on Days 1, 8, 15, 29, 57, and 71; anytime during visit on Days 3, 74, 85, 106, 127, and 155]		
			(4) Number of Participants With Any AE, SAE, and Adverse Events of Special Interest (AESI) [Time Frame: Up to 22 weeks]		
		NCT03285191	Number of subjects with RA participating in CE interviews [Time Frame: 1 day]		
MORAb-022	GM-CSF	NCT01357759	Safety measures to include adverse events, clinical laboratory results, vital signs, ECGs, physical examinations, local tolerability at the infusion site single escalating intravenous (IV) doses of MORAb-022 in healthy subjects and subjects with RA [Time Frame: Approximately 113 days]	Inhibiting M1 macrophage polarization	
Namilumab (AMG203)	GM-CSF	NCT01317797	(1) Number of Participants for Clinically Significant Clinical Laboratory Results, Clinically Significant Electrocardiogram (ECG) Findings, Clinically Significant Vital Signs, Clinically Significant Pulmonary Function Tests, and Clinically Significant Physical Examination Findings [Time Frame: From Day 1 Up to Day 118]	Inhibiting M1 macrophage polarization	(209, 210)
			(2) Number of Participants Reporting One or More Treatment Emergent Adverse Events [Time Frame: From Day 1 Up to Day 118]		
		NCT02393378	Change From Baseline in Synovitis, Erosion and Bone Marrow Edema (Osteitis) Score at Week 24 [Time Frame: Baseline and Week 24]		
		NCT02379091	Change From Baseline in DAS28-CRP at Week 12 [Time Frame: Baseline and Week 12]		
Mavrilimumab	GM-CSFRα	NCT00771420	Incidence and severity of adverse events • Changes in vital signs, ECG, lung function tests and clinical laboratory values [Time Frame: End of study]	Inhibiting M1 macrophage polarization	(211–216)
		NCT01050998	(1) Percentage of Participants Who Achieved DAS28-CRP Response at Day 85 [Time Frame: Day 85]		
			(2) Percentage of Participants Who Achieved DAS28-CRP Response at Day 85 by Region [Time Frame: Day 85] … etc. (24 items in total)		
		NCT01706926	(1) Change From Baseline in DAS28-CRP Score at Day 85 [Time Frame: Baseline and Day 85]		
			(2) Percentage of Participants Who Achieved ACR20 Responses at Day 169 [Time Frame: Day 169]		
		NCT01715896	(1) Percentage of Participants Who Achieved ACR20 Responses, ACR50 Responses, and ACR70 Responses at Day 169 [Time Frame: Day 169]		
			(2) Percentage of Participants Who Achieved DAS28-CRP Response at Day 169 [Time Frame: Day 169]		
			(3) Percentage of Participants Who Achieved Health Assessment Questionnaire Disability Index (HAQ-DI) Score Improvement From Baseline and ≥0.25 at Day 169 [Time Frame: Day 169]		
		NCT01712399	(1) Number of Participants With Treatment-Emergent Adverse Events (TEAEs) and Treatment-Emergent Serious Adverse Events (TESAEs) [Time Frame: From the start of drug administration up to 12 weeks after the last dose for the study (approximately up to 3 years)]		
			(2) Number of Participants With Clinical Laboratory Abnormalities Reported as TEAEs [Time Frame: From the start of drug administration in the study up to 12 weeks after the last dose (approximately up to 3 years)] … etc. (10 items in total)		

*NA, Not applicable

Anti-TNF-α therapy is currently the most widely used targeted biological agent. As mentioned above, sICAM1 may represent the myeloid subtype in synovial tissue and predict the therapeutic response to TNF-α therapy. Research has shown that lymphoid aggregates in synovial tissue are an independent negative predictive factor for anti-TNF-α therapy (66). A prospective study of the effect of infliximab on lymphoid aggregates after 16 weeks of treatment in RA patients, consistent with the above, also showed that patients with lymphocyte aggregates show a lower treatment response (217). By examining the expression profile of infliximab in the treatment of RA by arthroscopy, it was found that high-level tissue inflammation responds better to anti-TNF-α therapy (218). An evaluation of the effect of anti-TNF-α monoclonal antibody cA2 on RA showed that cA2 significantly reduced the migration of neutrophils, the number of infiltrated immune cells, and the expression levels of IL-8 and MCP-1, which relieve synovial inflammation (219). RA patients were injected with 10 mg/kg infliximab and underwent synovial biopsy. The results showed that the extents of ACR20, ACR50, and TNF-α, IL-1α, and IL-1β synthesis were all reduced (220). Studies have shown that high resistin is associated with high disease inflammation in RA and can be used as a biomarker to distinguish the early activity of RA from the increased risk of erosive diseases and to predict the treatment response of DMARD and infliximab (203). A clinical study predicting the response of RA to infliximab by measuring biochemical, immune, and bone markers in serum and using transcriptome analysis to identify gene expression characteristics in PBMCs is currently ongoing (NCT00213564). In addition, a randomized, multi-center biological experiment predicting the biomarkers of the effects of four biological agents (infliximab, tocilizumab, rituximab, abatacept) on RA (NCT01638715) has been coordinated. Similar clinical trials should be designed to predict the treatment response of different treatment modalities and to screen appropriate biomarkers. Baseline DAS28-CRP can predict the response of early RA patients to MTX treatment, and when MTX monotherapy fails, adding another TNF-α inhibitor, adalimumab, can achieve better clinical prognostic results (204). Another study determined the gene expression profile of RA patients with poor response to adalimumab and found 439 marker genes, primarily related to cell division and immune regulation pathways. The high expression levels of *IL-7R*, *CXCL11*, *IL-18*, *IL- 18 receptor accessory (IL-18rap)*, and *MK167* were related to absence of response to adalimumab treatment in the synovial membrane (221).

Possible indications for using rituximab in RA, including poor response to anti-TNF-α therapy, associated lymphoproliferative disease, RA that overlaps with systemic lupus erythematosus, Sjogren’s syndrome, and undifferentiated connective tissue disease have previously been reviewed (222). Given the heterogeneity of cells expressed in the pathological model of synovial tissue, among which autoimmune lymphoid B cells play an important role, the importance of targeting B cells to treat RA is self-evident. Rituximab can cause CD20^+^ B cell depletion. Some clinical studies have found that the use of rituximab alone or in conjunction with MTX has a higher rate of patient recovery than MTX alone (223, 224). The study found that a normal level of CD19^+^B cells and an increase in CD19^+^CD27^-^lgD^-^double negative B cells can predict RA patients’ response to rituximab (197). Serum IL-33 can be used as a predictor of response to rituximab in treating RA independently or in combination with RF or anti-CCP and high serum IgG (198, 199). Elevated levels of IgM-RF, IgG-RF, IgA-RF, anti-CCP, and CRP increase the sensitivity of RA patients with poor anti-TNF-α responses to rituximab response prediction (202). Biopsy analysis of the synovial tissue of RA patients receiving rituximab showed that DAS scores, RF, ACPA, T cells, macrophages, peripheral blood, and synovial B cells were significantly reduced after the treatment; the reduction of plasma cells indicated the therapeutic effect of rituximab (225). CXCL13 is a B cell chemokine and a serum biomarker of RA. By detecting the levels of B cells, CXCL13, RF-IgM, and anti-CCP in 20 RA patients treated with rituximab for six months, it was found that patients with detectable B cells had higher levels of CXCL13 and RF-IgM than those with undetectable B cells. The synovial tissue with high expression level of serum CXCL13 has increased levels of IL-1β, IL-8, MMP-1, and MMP3, suggesting that the level of serum CXCL13 can be used as a predictor of B cell depletion therapy in RA. In addition, the expression of CXCL13 is related to many markers of synovitis (74). CCL19 is also a B cell chemokine. Studies have shown that the serum levels of CXCL12, CXCL13, and CCL19 are increased and positively correlated with a variety of B cell markers, such as serum immunoglobulin IgG, IgA, BAFF, free light chain, and RF. It can also be used as a predictor of the response to rituximab (200). In addition, studies of rituximab treatment have shown that the low frequency of CD27^+^ memory B cells may be more beneficial in the B cell-driven RA subtypes (201). Studies have found that elevated levels of IgM-RF, IgG-RF, IgA-RF, anti-CCP, and CRP increase the sensitivity of prediction to rituximab therapy for RA patients who have a poor response to anti-TNF-α therapy (202). DAS28 scores, IgM-RF, anti-ACPA, synovial B cells, synovial T cells, and synovial macrophages were significantly reduced after rituximab treatment (225). Rituximab almost completely exhausted the number of circulating B cells in RA patients who failed anti-TNF-α therapy, and all achieved varying degrees of ACR response, which was accompanied by a decrease in synovial immunoglobulin synthesis (226). Another study used high-throughput PCR to detect the clinical response to rituximab in RA and found that the high expression level of inflammation-related genes in T cells and macrophages in the synovium were appropriate predictors for treatment response. The higher expression levels of interferon-α and remodeling-related genes indicated a poor response to rituximab (70). Combined with another clinical study to evaluate the therapeutic characteristics of rituximab on lymphatic T cells in RA patients, it was found that rituximab accidentally depleted a large number of CD4+ T cells, implying that B cells, T cells, and macrophages are interrelated in RA (196). Two other clinical trials to observe rituximab in RA patients with insufficient response or intolerance to anti-TNF-α therapy are underway, awaiting the release of the corresponding results (NCT01592292, NCT02079532). Also, RA may accelerate the progression of atherosclerosis, and the TNF-α inhibitor can alleviate atherosclerosis by regulating free radicals and endothelial dysfunction, involving the regulation of vascular endothelial growth factor and endothelial nitric oxide synthase (227, 228).

GM-CSF promotes monocyte differentiation into M1 pro-inflammatory macrophages in RA. A randomized, multi-center clinical trial for RA has revealed that MOR103, a human monoclonal antibody against GM-CSF, is well tolerated, safe, and effective for patients with RA. Patients in the 1.0 and 1.5 mg/kg MOR103 groups had significantly improved DAS28 and joint counts; the 1.0 mg/kg dose was the best correlated with the reduction in disease severity (208). A phase 1b, randomized clinical study evaluated the safety and tolerability to receive AMG203, a GM-CSF ligand with a high-affinity monoclonal antibody, after MTX treatment for ≥12 weeks and revealed that 150 mg or 300 mg of AMG203 exhibited good tolerance and improvement of disease activity indicators, such as DAS28-CRP (209). A phase II randomized, double-blind, placebo-controlled clinical trial studied the efficacy of AMG203 (20, 80, or 150 mg) in RA patients with insufficient response to MTX or anti-TNF antibody treatment. The results showed that the DAS28-CRP level for 12 weeks with AMG203 150 mg was significantly reduced at baseline. However, serious adverse events (myocardial infarction) occurred without a dose–response relationship (210). By evaluating the response of a total of 1145 RA patients in five studies with mavrilimumab, a human monoclonal antibody against GM-CSF receptor (GM-CSFR)-α, it was found that DAS28-CRP was significantly reduced after 12 weeks of treatment, and there were no significant adverse events, indicating good treatment efficacy and tolerability (229). The therapeutic intervention effect for ACR20 response to mavrilimumab 150 mg + MTX, mavrilimumab 100 mg + MTX, tofacitinib 10 mg + MTX, and tofacitinib 5 mg + MTX on RA patients with insufficient DMARD response was successively reduced, among which mavrilimumab 150 mg + MTX and mavrilimumab 100 mg + MTX had the most significant ACR20 response (230). In another study, mavrilimumab and golimumab, an anti-TNF monoclonal antibody, were combined with MTX to treat RA patients with insufficient DMARD response. Evidently, the ACR20, ACR50, and ACR70 response rates were not significantly different, which may be due to the non-optimal therapeutic dose (mavrilimumab 100 mg + MTX). Although there was no direct comparison between the two therapies, the effect of mavrilimumab is clinically promising (211). Similar research results showed that mavrilimumab could reduce the DAS28-CRP of RA patients and achieve the ACR20 remission rate, both of which indicate the potential therapeutic effect of mavrilimumab for the inhibition of subsets of monocytes and macrophages in patients with RA (212–215).

Increased IL-6 activity in patients with RA is significantly related to synovial inflammation and autoantibody production. As mentioned above, CXCL13 is used as a biomarker to predict the efficacy of anti-IL-6R therapy (tocilizumab) in RA with a synovial lymphoid phenotype. An open-label, multi-center, prospective, single-arm study used hand-foot radiography and hand magnetic resonance imaging to detect the efficacy of a tocilizumab subcutaneous injection for 24 weeks on joint damage in RA patients. The results showed synovial inflammation, the DAS28 score was lessened, and the treatment was well-tolerated (195). By comparing the gene expression data of tocilizumab, MTX, rituximab, and adalimumab in synovial biopsy tissue of RA patients, it was shown that IL-6R, chemokines, and T cell activation-related gene expression level was reduced after treatment with tocilizumab, and showed a similar gene expression pattern to that of treatment with rituximab and MTX, which implies that B cells and IL-6 have a molecular synergistic effect (231). In addition, after tocilizumab treatment of RA patients, the ERK in the synovial tissue was significantly increased, the number of lymphatic B cells (CD20) was significantly reduced, and IL-6 was blocked entirely, which was comparable to MTX treatment (232). Another study on 65 RA synovial biopsy tissues found that TNF-α-induced disease activity-related transcripts were highly enriched in patients with early RA, which is associated with higher disease activity and predicts a poor response to other therapies, such as MTX and tocilizumab (233). BAFF protects autoimmune B cells by binding to APRIL. Using 30 mg and 80 mg doses of tabalumab (anti-BAFF monoclonal antibody) to treat patients with insufficient TNF-α therapy, some therapeutic effects were observed earlier, including changes in ACR20, ACR50, and DAS28-CPR (205). Spebrutinib (CC-292), a Bruton’s tyrosine kinase inhibitor, can inhibit B cell proliferation and osteoclast production *in vitro* and can reduce the number of CD19B cells, serum CXCL13, macrophage inflammatory protein-1β (MIP-1β), carboxy-terminal collagen cross-linking telopeptide, and C-telopeptide of type I collagen (CTx-1), among which CD19B cells and CTx-1 can predict the response of RA patients to spebrutinib (234). Tofacitinib, a JAK inhibitor, can inhibit the angiogenesis and migration ability of endothelial cells (ECs) *in vitro* and reduce VEGF levels in CIA mice, suggesting that its efficacy could be related to angiogenic inhibition (235). A randomized, double-blind, phase II clinical trial tested the effect of 10 mg of tofacitinib twice daily on patients with poor MTX response for 28 days. The results showed that the gene expression level in the synovial membrane is decreased with the well EULAR response, such as the reduction of *MMP-1*, *MMP-3*, *CCL2*, *CXCL10*, and *CXCL13* levels, which may be related to the decrease in phosphorylation of STAT1 and STAT3 (206). The mechanism of abatacept is to block cytotoxic T lymphocyte associated antigen 4 (CTLA4) to prevent T cell costimulatory signals and T cell activation. A prospective, multi-center, observational clinical trial studied the efficacy of abatacept in elderly RA patients and found that after abatacept treatment, more patients improved compared with DMARD. Compared with patients with no response or poor response to DMARD, abatacept was more effective (UMIN000014913) (236). After abatacept treatment, no significant changes in synovial immune infiltration were found, but the expression levels of pro-inflammatory genes (IFN-γ and IL-1β), MMP-1, and MMP-3 was significantly reduced (237). Several other biological agents (anakinra, ustekinumab, and guselkumab) are targeted at IL-1R, IL-12/IL-23, and IL-23, respectively, and several related clinical trials are in progress (207).

## Conclusions

Although various treatment options exist for RA, many of these options show little to no efficacy, even when used in combination. Understanding the heterogeneity in the cellular and molecular features of RA could aid the development of safe and effective treatments. Many clinical studies on RA are underway; however, it remains unclear how to accurately find biomarkers that predict the patient’s response to a certain therapy. Finding such biomarkers is a major goal of precise treatment. Treatment should be combined with procedures like synovial biopsy and modern techniques such as arthroscopic surgery, assisted computer imaging, and multi-omics to better evaluate the disease activity/progression and the patient’s response. Inflammation in RA is a complex process and not an independent and discrete set of events. There are typically multiple synovial tissue disease expression patterns in patients with RA with many different subsets of corresponding cells. One of these subsets may be dominant at a specific disease stage; however, it may not appear independently. Well-designed clinical trials that explore the impact of various cell subgroups on different RA therapies need to be urgently conducted for clinical application. The goal is to support the beneficial cell subsets and to inhibit the cell subsets that hinder a specific treatment. A certain strategy may inhibit the activity of a cytokine but is typically accompanied by serious adverse reactions possibly related to the breakdown of the physiological balance of cytokines. Therefore, targeting the activity of common mediators of multiple cells and maintaining the corresponding balance based on disease activity and biomarker response is essential for drug therapy development. 

## Author Contributions

JZ is responsible for the collection, collation, and writing of the original manuscript. SG, SS, and DH are responsible for the concept development, revision, and review of the manuscript. All authors contributed to the article and approved the submitted version.

## Funding

This work was funded by the National Natural Science Funds of China (81774114), Shanghai Chinese Medicine Development Office, Shanghai Chinese and Western Medicine Clinical Pilot Project (ZY(2018–2020)-FWTX-1010), Shanghai Chinese Medicine Development Office, Shanghai Traditional Chinese Medicine Specialty Alliance Project (ZY(2018-2020)-FWTX-4017), National Administration of Traditional Chinese Medicine, Regional Chinese Medicine (Specialist) Diagnosis and Treatment Center Construction Project-Rheumatology.

## Conflict of Interest

The authors declare that the research was conducted in the absence of any commercial or financial relationships that could be construed as a potential conflict of interest.

## Publisher’s Note

All claims expressed in this article are solely those of the authors and do not necessarily represent those of their affiliated organizations, or those of the publisher, the editors and the reviewers. Any product that may be evaluated in this article, or claim that may be made by its manufacturer, is not guaranteed or endorsed by the publisher.

## Glossary

**Table:**

RA	rheumatoid arthritis
DMARDs	disease-modifying anti-rheumatic drugs
TNF-α	tumor necrosis factor-α
RF	rheumatoid factor
ACPA	anti-citrullinated protein antibodies
FLS	fibroblast-like synoviocyte
MLS	macrophage-like synoviocyte
CPJ	cartilage-pannus junction
MMP	matrix metalloproteinase
NOD	nucleotide-binding oligomerization domain
NF-κB	nuclear factor kappa-light-chain-enhancer of activated B cells
IL	interleukin
CXCL	chemokine CXC ligand
CCL	CC-chemokine ligand
IL-6R	IL-6 receptor, among others
sIL-1R1	soluble IL-1 receptor type 1, among others
TGF	transforming growth factor
BMP	bone morphogenetic protein
SMAD	Sma Mothers Against Decapentaplegic
sICAM-1	soluble intercellular adhesion molecule-1
MC	mast cell
MCP-1	monocyte chemoattractant protein-1
VEGF	vascular endothelial growth factor
FGF-2	fibroblast growth factor-2
CMP	common myeloid progenitor
BM	bone marrow
MPS	mononuclear phagocyte system
MTX	methotrexate
IC	IgG-containing immune complexes
APRIL	a proliferation-inducing ligand
TLR	toll-like receptor
ENO-1	alpha-enolase-1
ENOSF1	enolase superfamily member 1
tmTNF	transmembrane TNF
Mo-DC	monocyte-derived dendritic cell
mDC	myeloid DC
pDC	plasmacytoid DC
BAFF	B cell activating factor
anti-CCP	anti-cyclic citrullinated peptide
AA	adjuvant-induced arthritis
PAR2	protease-activated receptor-2
LPM	large peritoneal macrophage
SPM	small peritoneal macrophage
SM	synovial macrophage
ESM	embryonic SM
BMSM	bone marrow-derived SM
MRP	myeloid related proteins
IRF	interferon regulatory factor
CIA	collagen-induced arthritis
PGE2	prostaglandin E2
MAPK	mitogen-activated protein kinase
VCAM1	vascular cell adhesion protein 1
Tfh	T follicular helper
GM-CSF	granulocyte-macrophage colony-stimulating factor
GM-CSFR	GM-CSF receptor
CTx-1	C-telopeptide of type I collagen
EC	endothelial cell
CTLA4	cytotoxic T lymphocyte associated antigen 4
BTK	Bruton’s tyrosine kinase
SNP	single nucleotide polymorphism
SPAG16	sperm-associated antigen 16
GWAS	genome-wide association study
DREAM	dialogue on reverse engineering assessment and methods
RANKL	receptor activator of nuclear factor-κB ligand
PTPN22	protein tyrosine phosphatase nonreceptor 22
CCR6	C-C chemokine receptor 6
PADI4	peptidyl arginine deiminase type IV
STAT4	signal transducer and activator of transcription 4 protein
CTLA4	cytotoxic T-lymphocyte antigen 4
SNPs	single nucleotide polymorphisms
NSAIDs	nonsteroidal anti-inflammatory drugs
mTSS	modified total sharp score
ITT	intention to treat
SPS	standard population set
HAQ-DI	HAQ disability index
AUC	area under the curve
SDAI	simplified disease activity index
AEs	adverse events
SAEs	serious adverse events
AESI	adverse events of special interests
C_max_	maximum observed concentration
AUC_[0-t]_	AUC from time zero to the time
AUC_[0-inf]_	AUC from time zero extrapolated to infinity
AUC_tau_	AUC over the dosing interval
T_max_	time to reach C_max_
t_1/2_	terminal half-life
ECG	electrocardiogram
TEAEs	treatment-emergent adverse events
TESAEs	treatment-emergent serious adverse events
